# Dual-Cameras-Based Driver’s Eye Gaze Tracking System with Non-Linear Gaze Point Refinement

**DOI:** 10.3390/s22062326

**Published:** 2022-03-17

**Authors:** Yafei Wang, Xueyan Ding, Guoliang Yuan, Xianping Fu

**Affiliations:** School of Information Science and Technology, Dalian Maritime University, Dalian 116026, China; wangyafei@dlmu.edu.cn (Y.W.); dingxueyan@dlmu.edu.cn (X.D.); fxp@dlmu.edu.cn (X.F.)

**Keywords:** driving environment, gaze tracking, non-linear refinement

## Abstract

The human eye gaze plays a vital role in monitoring people’s attention, and various efforts have been made to improve in-vehicle driver gaze tracking systems. Most of them build the specific gaze estimation model by pre-annotated data training in an offline way. These systems usually tend to have poor generalization performance during the online gaze prediction, which is caused by the estimation bias between the training domain and the deployment domain, making the predicted gaze points shift from their correct location. To solve this problem, a novel driver’s eye gaze tracking method with non-linear gaze point refinement is proposed in a monitoring system using two cameras, which eliminates the estimation bias and implicitly fine-tunes the gaze points. Supported by the two-stage gaze point clustering algorithm, the non-linear gaze point refinement method can gradually extract the representative gaze points of the forward and mirror gaze zone and establish the non-linear gaze point re-mapping relationship. In addition, the Unscented Kalman filter is utilized to track the driver’s continuous status features. Experimental results show that the non-linear gaze point refinement method outperforms several previous gaze calibration and gaze mapping methods, and improves the gaze estimation accuracy even on the cross-subject evaluation. The system can be used for predicting the driver’s attention.

## 1. Introduction

Monitoring a driver’s attention is one of the most vital components of advanced driver assistance systems (ADASs) which monitor the driver’s status and prevents traffic accidents from happening when the driver is distracted [1,2,3,4]. According to the demand for safe driving, drivers should maintain sufficient awareness in order to take a series of actions during the whole driving process. Distraction is always facilitated by driving over long durations under monotonous conditions or otherwise being “lost in thought”, which could decrease the driver’s awareness and impair their reaction speed with regard to regaining control of the vehicle. A better understanding of driver behaviors through driver attention analysis could promote ADAS to generate optimal control strategies corresponding to the current driving situation.

Eye gaze is often closely related to driving behaviors and is treated as a vital cue for detecting the driver’s visual attention. Many remote gaze-tracking-based techniques, which are based on head and eye pose features extracted from driver videos, have popularly been used in driving scenarios due to their high user friendliness. Gaze mapping is the essential step to build the mapping relationship between the driver’s eye gaze features and the corresponding gaze location for gaze prediction inside or outside the vehicle. Manual gaze mapping methods necessarily require driver compliance [5,6] and human cooperation before each session, which adds significant inconvenience for driver eye gaze tracking systems. Several automatic mapping methods have approached driver gaze zone estimation utilizing gaze behavior or gaze frequency on specific driving data [7,8,9], which are heavily dependent on the parameter estimation accuracy and fragile to the outliers. This makes the driver gaze estimation system session-exclusive or person-exclusive, and requires re-calibration or re-mapping in the setup procedure before each session, which seriously affects the applicability of the pre-trained gaze model. However, there is little progress that works on such intrinsic problems in the driver’s gaze estimation system.

This paper presents a domain knowledge-based solution to mapping and tracking the driver’s predicted eye gaze points to their real location in the naturalistic dual-cameras-based monitoring system. The driver’s natural mirror-checking actions are used for finding representative gaze points in the context, which are later generated by progressive clustering in an automatic manner. The representative gaze points can be treated as known gaze points for gaze point refinement. The non-linear gaze point refinement treats these representative gaze points as known gaze points, builds simple and straightforward re-mapping for gaze estimation, eliminates the estimation bias related to the session, and makes the pre-trained gaze model more generalized. The main contributions are as follows:

- A dual-cameras-based driver eye gaze tracking system using non-linear gaze point refinement is presented for deploying a pre-trained supervised gaze model in the unconstrained environment. This method makes an initial attempt to reduce the estimation bias in separate model training. It increases the flexibility of system setup and does not require any human intervention.
- An effective gaze point non-linear global refinement with two-stage clustering is presented to extract the typical gaze points by maximizing fixation possibilities. This method aligns the initial unknown gaze points to specific calibration points by topology preservation. It is person-independent and can be directly utilized as post-processing for many pre-trained gaze models.
- Experimental results of real driving scenarios demonstrate that the proposed method reduces the gaze estimation error of the pre-trained model and even has a better performance on cross-subject evaluations. It can be used as a simple-but-effective baseline method for driver gaze calibration or gaze mapping.

The rest of this paper is organized as follows. In Section 2, the driver eye gaze prediction and gaze calibration works are introduced. Section 3 describes the details of the proposed driver eye gaze tracking system. In Section 4, several experimental results and error analysis are given. Section 5 presents a brief conclusion of the proposed system.

## 2. Related Works

The human head and eye dynamics are fundamental to revealing the drivers’ gaze points that represent their current visual attention. Therefore, it has been widely used to detect the visual distraction and understand driver behaviors by exploiting the driver’s head and eye orientation [10]. In early works, several methods and devices were approached for accurate gaze information in driving environments, such as head-mounted eye trackers. These trackers are intrusive and costly, and might change driving habits and behavior. With the advances of the remote driver eye gaze tracking techniques, non-intrusive systems based on computer vision have been well applied due to their user-friendliness [11].

### 2.1. Driver’s Eye Gaze Estimation

#### 2.1.1. Feature-Based Systems and Appearance-Based Systems

In vision-based driver behavior monitoring systems, it is acceptable for the driver’s head orientation regarding as coarse gaze direction [12]. Through a gaze zone estimator, the probability of the driver gaze direction could be obtained. These estimators classify the gaze points into partitioned gaze zones that are inside or outside the vehicle, such as side mirrors, rear-view mirror and windshields. Most of the driver gaze estimation works build the estimator with facial appearance [13,14], head movement [15,16,17,18], or eye movement [19,20] in a monocular camera system.

Wang et al. [21] estimated the driver’s head pose in the depth image by a point-cloud alignment-based approach, and computed the eye direction in the RGB image by appearance-based gaze estimation approach. In this work, the author determined the gaze zone results by the weighted sum of the head and eye direction. Jha et al. [22] used the head position and orientation as the input features and built the estimator via Gaussian process regression (GPR). Lundgren et al. [23] applied both head features and eye features to train the estimator via the GPR method.

#### 2.1.2. Deep Learning-Based Systems

With the vigorous development of deep learning technology, various attempts have started being made to achieve eye gaze estimation in the driving environment. All these deep learning-based methods consist of a facial landmark detection or feature extraction module connected to the model input. The network is used to fuse the high-dimensional rich features. Yu et al. [24] fused appearance-based features and geometric information in the convolutional neural networks (CNNs) for efficient driver gaze prediction. Lyu et al. [25] trained the CNN model for gaze classification. This work carried out a fusion network that combined two classification networks. Lollett et al. [26] combined face detection, facial landmarks detection, eye image post-processing in one system, and classified the extracted feature vector into the driver’s gaze zone. They tested the model’s robustness in several challenging driving scenarios, such as non-uniform illumination and face occlusions.

For the dual-cameras-based system, the gaze points are predicted on the dynamic scene, not just the interior components of the vehicle. The face camera is often fixed at the windshield or center console to capture the driver’s video in the field of view. The scene camera is installed behind the driver to mimic the driver’s view and shows the gaze points in a naturalistic driving environment. Most current driver gaze estimation research studies have evaluated the predicted accuracy of gaze location regression or gaze zone classification inside or outside the vehicle. These studies are based on the domain knowledge in the training dataset and build the gaze model beforehand. It is vulnerable and inapplicable to the uncertain and complex on-road driving environment. Therefore, the driver’s gaze tracking system needs a more flexible setup for the subjects about looking by head movements and eye movements.

### 2.2. Driver’s Eye Gaze Calibration

Gaze calibration is a significant step in the gaze tracking system, which is employed to build the relationship between the human eye and the gaze location. The gaze calibration of driving scenes is usually realized by using typical characteristics of the scene, such as common components inside the vehicle, or the driving behavior characteristics of the driver. Some methods bring the calibration features of the indoor scene as additional prior information.

Fu et al. [7] introduced a calibration method for head pose estimation that regards the common gaze zones as the calibration points, such as the mirror gaze zone and the instrument board gaze zone. This method was realized by a self-learning algorithm, and its parameters can be periodically updated. Through the solving of the relevant coefficients, it refined the predicted head pose to the real head pose. Yamashiro et al. [8] used less gaze zone related to the driving behavior as calibration points under specific lane-changing conditions such as the rear-view mirror gaze zone, one-side mirror gaze zone, and the forward gaze zone. This method assumes that the data clustering centers should be the calibration points due to the possibility. This established the mapping relationship through the transformation of the three calibration points. Yoon et al. [27] introduced a stereo-camera-based calibration method using one calibration point. This method takes full advantage of the driver’s prior information collected in the desktop monitor. Yang et al. [5] utilized a non-linear polynomial method to build the mapping relationship linking the features to the gaze points. Yuan et al. [9] used six gaze zones related to driver gaze glance as calibration points. This extracted and clustered more representative gaze features to build the regression by GPR. Due et al. [6] established the mapping between the driver view and road view by I-DGAZE model, but the status of the drivers was still varying and needed to be fine-tuned by driver-specific gaze calibration. Their results showed that the gaze mapping or gaze calibration can greatly reduce the error of the trained CNN model.

It is worth noting that the gaze classification accuracy varies significantly between drivers and that the person-specific driver gaze estimation system obviously makes sense. To the best of our knowledge, few studies have aimed to implement the gaze mapping system with person-specific gaze point refinement for a naturalistic driving environment.

## 3. Proposed Method

The proposed method contains three modules: driver status tracking, pre-trained gaze model, and non-linear gaze point refinement. For the dual cameras system, the driver status tracking module infers the status features from the face camera, and the pre-trained gaze model module maps the features to the gaze points on the scene camera.

In the driver status tracking module, all the relevant driver status is defined and initialized. The status contains both the signals of the head movement and the eyes movement. Since the observed measurements have random disturbances to a certain extent, the Kalman filter is adopted to track and update the measurements. In the pre-trained gaze model module, the corresponding gaze mapping from the driver status features to the gaze points is built during the offline training. The model is then used to give the initial gaze points. At this point, the initial driver gaze tracking system has been built.

In the non-linear gaze point refinement module, the initial gaze points is corrected to the real locations. Firstly, the candidate gaze points are automatically extracted by two-stage gaze point clustering. The forward gaze zone center is measured by density peak clustering. The candidates of the three mirror gaze zones are selected from the maximum distance points on the time sample fragments. All possible gaze zone centers are found by Gaussian mixture cluster, and the parameters are updated by the expectation-maximization algorithm in each gaze zone. Then, based on the known mapping relationships of the candidate gaze points, a simple non-linear method is adopted to further adjust the gaze points on the image plane.

**Figure 1:**
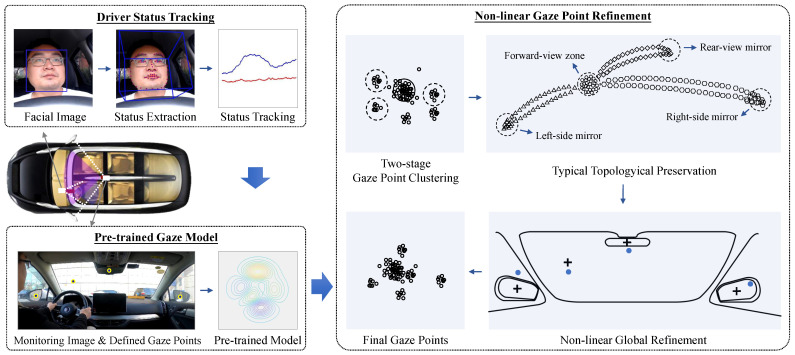
Overview of the proposed system.

The whole framework is running automatically without any manual interpretation. Within it, the non-linear gaze point refinement module periodically refines and updates the gaze mapping parameters, making the framework itself an automatic system. Figure 1 illustrates the proposed system. This section describes each module of the system.

### 3.1. Driver Status Tracking

This paper uses a single camera to achieve the signal acquisition of the driver. Since head position, head rotation, and eye rotation all contribute to driver gaze estimation, this paper considers all these signals in the driver status model. This is consistent with the driver’s eye gaze glance in real driving scenarios, as drivers usually move their head and eyes to focus on the target. To perform the prediction step, the Unscented Kalman filter is employed.

In this paper, the driver state $x_{k}$ that contains information about the driver’s head position, head rotation, and gaze direction is introduced as the indicator of the driver’s visual attention. The properties in the state features make a continent way to determine the driver’s eye gaze if they were obtained. In practice, the state $x_{k}$ is divided into three components: head state; left eye state; and right eye state. $x_{k}={\left[h_{k}^{T},e_{k,l}^{T},e_{k,r}^{T}\right]}^{T}$, where:

(1) $$\begin{array}{c} h_{k}={\left[x_{k},y_{k},z_{k},h_{k}^{\alpha },h_{k}^{\beta },h_{k}^{\gamma },{\dot{x}}_{k},{\dot{y}}_{k},{\dot{z}}_{k},{\dot{h}}_{k}^{\alpha },{\dot{h}}_{k}^{\beta },{\dot{h}}_{k}^{\gamma }\right]}^{T},\\ e_{k,l}={\left[e_{k,l}^{\alpha },e_{k,l}^{\beta },e_{k,l}^{\gamma },{\dot{e}}_{k,l}^{\alpha },{\dot{e}}_{k,l}^{\beta },{\dot{e}}_{k,l}^{\gamma }\right]}^{T},\\ e_{k,r}={\left[e_{k,r}^{\alpha },e_{k,r}^{\beta },e_{k,r}^{\gamma },{\dot{e}}_{k,r}^{\alpha },{\dot{e}}_{k,r}^{\beta },{\dot{e}}_{k,r}^{\gamma }\right]}^{T}. \end{array}$$

The head position and head rotation are denoted by ${\left[x_{k},y_{k},z_{k}\right]}^{T}$ and ${\left[h_{k}^{\alpha },h_{k}^{\beta },h_{k}^{\gamma }\right]}^{T}$. Furthermore, their corresponding change rates are denoted by ${\left[{\dot{x}}_{k},{\dot{y}}_{k},{\dot{z}}_{k}\right]}^{T}$ and ${\left[{\dot{h}}_{k}^{\alpha },{\dot{h}}_{k}^{\beta },{\dot{h}}_{k}^{\gamma }\right]}^{T}$. The eye gaze direction of both the left eye and the right eye is independent from the head rotation and is denoted by ${\left[e_{k,l}^{\alpha },e_{k,l}^{\beta },e_{k,l}^{\gamma }\right]}^{T}$ and ${\left[e_{k,r}^{\alpha },e_{k,r}^{\beta },e_{k,r}^{\gamma }\right]}^{T}$. The change rates of the two eye rotations are given as ${\left[{\dot{e}}_{k,l}^{\alpha },{\dot{e}}_{k,l}^{\beta },{\dot{e}}_{k,l}^{\gamma }\right]}^{T}$ and ${\left[{\dot{e}}_{k,r}^{\alpha },{\dot{e}}_{k,r}^{\beta },{\dot{e}}_{k,r}^{\gamma }\right]}^{T}$. The eye model is the same for the left eye and the right eye, and the gaze directions are the relative eye-in-head rotations. Therefore, the eye movement is independent from the head movement, and this property is useful for the gaze estimation process.

In this paper, the corresponding measurement vectors are given as $z_{k}^{g}=\left[z_{k}^{h},z_{k,l}^{e},z_{k,r}^{e}\right]$, where,

(2) $$\begin{array}{c} z_{k}^{h}=\left[x_{k},y_{k},z_{k},h_{k}^{\alpha },h_{k}^{\beta },h_{k}^{\gamma }\right],\\ z_{k,l}^{e}=\left[e_{k,l}^{\alpha },e_{k,l}^{\beta },e_{k,l}^{\gamma }\right],\\ z_{k,r}^{e}=\left[e_{k,r}^{\alpha },e_{k,r}^{\beta },e_{k,r}^{\gamma }\right]. \end{array}$$

#### 3.1.1. Process Model

This paper uses the following head model and eye model as the process models for the information processing. In practice, the driver’s head and eyes correspond to the camera coordinate system, respectively. Hence, the driver’s state can be expressed using the head status and eye status.

#### 3.1.2. Head Model

Inspired by Ref. [23], a constant velocity model is utilized in the head model. The current head pose is determined by the previous head pose, head change rate, and the model noise. Therefore, the head model is defined as

(3) $$h_{k}=A_{h}h_{k-1}+B_{h}v_{k-1},$$

where $v_{k-1}∼N\left(0,Q^{h}\right)$ is Gaussian model noise. A and B are stated as

(4) $$A_{h}=\left[\begin{array}{cc} I & T_{s}I\\ 0 & I \end{array}\right],B_{h}=\left[\begin{array}{c} \frac{T_{s}^{2}}{2I}\\ T_{s}I \end{array}\right],$$

where $T_{s}$ denotes the sampling time.

#### 3.1.3. Eye Model

The eye movement can generally be categorized into three types: fixation; saccades; and smooth pursuit. The eye model describes eye motion that either fixates on the target, quickly changes between fixation points, or smoothly follows the moving target. The correlation between the head movement and the eye movement is considered in the eye model. The previous method used the gaze signal as the combination of the head pose and gaze pose; here, in this paper, the eye rotation provided by the eye model is just the gaze pose. The model describes the eye rotation as the weighted sum of the current eye rotation and the eye rotation generated by the head model:

(5) $$e_{k}=\lambda_{e}e_{k}^{1}+\left(1-\lambda_{e}\right)e_{k}^{2},$$

where $0<\lambda_{e}<1$ is the distribution determined by the head model:

(6) $$e_{k}^{1}=A_{e}^{1}e_{k-1}^{1}+B_{e}^{1}v_{k-1},$$

where $v_{k-1}∼N\left(0,Q^{e}\right)$ is the Gaussian model noise and:

(7) $$A_{e}^{1}=\left[\begin{array}{c} T_{s}I\\ I \end{array}\right],B_{e}^{1}=\left[\begin{array}{c} \frac{T_{s}^{2}}{2I} \end{array}\right],$$

(8) $$e_{k}^{2}=A_{e}^{2}e_{k-1}^{2}+B_{e}^{2}v_{k-1},$$

where,

(9) $$A_{e}^{2}=\left[\begin{array}{c} 0\\ I \end{array}\right],B_{e}^{2}=\left[\begin{array}{c} \frac{T_{s}^{2}}{2I} \end{array}\right].$$

#### 3.1.4. Measurement Model

This part introduces the measurement model as the function of the driver state. As the process model, the measurement model consists of the head model and eye model.

Head Model: The head position and head rotation is measured in this paper, and the state vector in the measurement model is defined as

(10) $$z_{k}^{h}=Hh_{k}+w_{k}=\;\left[I\;0\right]h_{k}+w_{k}$$

where $w_{k}∼N\left(0,R^{h}\right)$ is the measurement noise.

Eye Model: The eye model uses the three-dimensional gaze direction as the state variable. The measurement model is stated as

(11) $$z_{k}^{e}=e_{k}+w_{k}$$

where $w_{k}∼N\left(0,R^{e}\right)$. When the observations are available in the measurement state, its measurement vector $z_{k}^{g}$ can be obtained.

### 3.2. Pre-Trained Gaze Model

In this paper, the traditional driver gaze estimation algorithm is used to train the gaze model when getting enough status samples in the dataset. Generally, the linear or non-linear model regresses the driver status features to specific gaze points on scene image. The pre-trained gaze model should not be over-fitted and has a balanced performance on the labelled training dataset and testing dataset. Mathematically, the gaze model can be written as

(12) $$\begin{array}{cc} \; & g_{x}=f_{x}\left(z_{1}^{g},z_{2}^{g},\ldots ,z_{k}^{g}\right)\\ \; & g_{y}=f_{y}\left(z_{1}^{g},z_{2}^{g},\ldots ,z_{k}^{g}\right) \end{array}$$

where $z_{k}^{g}$ denotes the *k*-th obtained status features in the dataset. $g_{x}$ and $g_{y}$ represent the gaze points in horizontal and vertical directions, respectively. $f_{x}\left(\cdot \right)$ and $f_{y}\left(\cdot \right)$ are the given linear or non-linear mappings of the gaze points. In this work, the gaze points are modeled in two independent directions.

It is worth noting that the robustness of the pre-trained gaze model obviously has a significant impact on the next step. If the prediction results of the pre-trained model are particularly poor, it is necessary to add much driver status features to accommodate the data diversity and train a new gaze model. Our goal is to refine the output of the model and make it easy to apply. At this point, it is also practical to build an online model as Ref. [9]. The refinement method works on the online model.

### 3.3. Non-Linear Gaze Point Refinement

In this section, the main processes of non-linear gaze point refinement are described.

#### 3.3.1. Two-Stage Gaze Point Clustering

**Figure 2:**
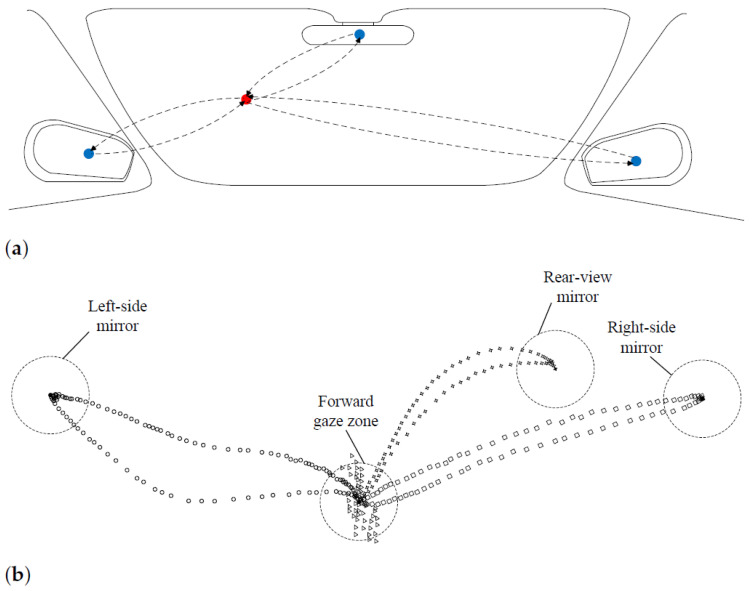
Typical eye glance allocation of mirror-checking behavior. (**a**) Schematic view of eye glance allocation. (**b**) Real-world examples in the gaze points space. Intuitively, the glance allocation for each mirror-checking behavior is discrete. The triangles denote the gaze points when the driver is facing the frontal area in the vehicle, and the circles, rectangles, and four-pointed stars denote the features’ trajectory when the driver turns to the left-side mirror, right-side mirror, and rear-view mirror, respectively. Here, the furthest feature points are used as the representative candidates for the driving behavior.

Mirror-checking behaviors can be regarded as typical eye glance allocation that benefits from both head and eye movement. The forward-view gaze zone is the major region of the driver’s visual attention, which means that the glancing of the mirror gaze zone goes back and forth from the forward-view gaze zone.Figure 2 gives an example of the mirror-checking behavior. It will be facilitated to the initial gaze points by supervised refinement. Previous works having studied the mirror gaze zones’ detection or classification include [28,29,30,31]. In this work, a two-stage gaze point clustering method was adopted to seek the gaze points of representative mirror gaze zones in continuous naturalistic driving data.

#### 3.3.2. Gaze Points Clustering

The driver maintains their visual attention on the road by moving their head and eye during the on-road driving, and uses the mirror gaze zones to assist their observation of the surrounding environment. Thus, most of the gaze points should be fixated on the forward-view gaze zone. This paper sets the center of the forward-view gaze zone as the global density center of the initial gaze points which is consistent with the actual situation. Considering the peak density measurement, the center has the characteristics of the largest value in the local neighbor density and the smallest value in the point distance. Hence, the local neighbor density and point distance can be computed by

(13) $$\begin{array}{c} \rho_{k}=\sum_{k^{\prime }}sgn\left(d_{kk^{\prime }}-d_{c}\right)\\ \delta_{k}=\min_{k^{\prime }\in \Omega -k}d_{kk^{\prime }} \end{array}$$

where sgn(·) denotes the signum function, its value equals 0, when *x* is negative; otherwise, its value equals 1. $d_{kk^{\prime }}$ means the distance between the *k*-th gaze point and $k^{\prime }$-th gaze point in the given space. $d_{c}$ represents the cutoff distance. Mathematically, the center of the forward-view gaze zone $e_{k}^{g}$ is the gaze point with $\max(\rho_{k})$ and $\min(\delta_{k})$. Its cluster density radius is given as $\tau =\frac{\lambda }{\left|\Omega \right|}\sum_{k^{\prime }\in \Omega -\bar{k}}d_{\bar{k}k^{\prime }}$, where |Ω| denotes the data size of the initial gaze points. λ means the statistical probability of the forward-view gaze zone.

#### 3.3.3. Mirror Gaze Points Clustering

The mirror gaze zones also have a higher local neighbor density, thence previous works directly clustered the forward-view gaze zone and mirror gaze zones in the same procedure. This matches the relatively dense distribution of the gaze points during long-term driving. However, the complexity and uncertainty make the exceptions inevitable. It is important to increase the effectiveness of the clustering results and improve the speed of the algorithm. Therefore, this paper filters out the candidate gaze points of the mirror gaze zone on the segmented data out of the forward-view gaze zone, and further use the Gaussian mixture clustering method to eliminate the noise.

The original gaze points data are converted to binary status data with directionality B. These status data indicate whether the gaze point is still in the forward-view gaze zone. When the data are out, its value is positive; otherwise, its value is negative. The data beyond a certain distance can be considered as those on mirror-checking action. Here, the binary status is defined as $B_{k}=sgn\left(d_{\bar{k}k^{\prime }}-\tau \right)$.

Mirror-checking behavior is a continuous action with processive time samples. The candidate gaze point for the single action can be the representative gaze point among the time samples. In this paper, the time samples are segmented into an importance fragment M. Hence, the candidate gaze points $e_{k}^{*}$ is computed as

(14) $$\begin{array}{c} g_{k}^{*}=\underset{g_{k}\in M}{\arg\max}\;d_{g_{\bar{k}}g_{k^{\prime }}} \end{array}$$

In this paper, the mirror gaze zones are assumed as the region with high local density and modeled by a Gaussian mixture model as single two-dimensional distribution for each mirror gaze zones. The gaze points of each gaze zone are i.i.d. with an underlying density of p(x). The finite mixture model p(x) is computed as

(15) $$p\left(x\right)=\sum_{m=1}^{M}\alpha_{m}N\left(g_{m}|\mu_{m},\Sigma_{m}\right)$$

where $\mu_{m}$ and $\Sigma_{m}$ are the parameters defined over the density or distribution. $\alpha_{k}$ are the mixture weights, $\sum_{m=1}^{M}\alpha_{m}=1$. Here, *M* is the components number as well as the number of the mirror gaze zone. In this scenario, the maximum likelihood is defined and estimated as follows:

(16) $$L_{ml}\left(\theta_{ml}\right)=\prod_{m=1}^{M}p\left(g_{m};\theta_{ml}\right)$$

where $\theta_{ml}=\left\{\mu_{1},\ldots ,\mu_{M},\Sigma_{1},\ldots ,\Sigma_{M}\right\}$. This cluster is solved by the expectation-maximization (EM) algorithm to proceed with a posteriori estimation of clustering parameters.

#### 3.3.4. Typical Topology Preservation

After obtaining the clustered gaze points, the main task is to assign the unsupervised gaze points with correct gaze zone labels and anchor positions. This paper assigns the clusters by typical topology preservation. As shown in Figure 2, the eye glance allocation of the mirror-checking behavior is similar in the gaze points space. At the same time, the motion of the initial gaze points holds the related position. Based on this observation, this paper assumes that the representative points of the cluster center has an approximate position that consists of the annotations on the image plane and the same motions with the head movement. In this work, the gaze point is uncalibrated and output by the pre-trained gaze model. Hence, the cost function is given as

(17) $$L_{tp}\left(\theta_{tp}\right)=\min\sum_{j}\cos^{-1}\frac{\left(g_{j}-g_{1}\right)\;\cdot \;\left(h_{j}-h_{1}\right)}{\left|g_{j}-g_{1}\right|\left|h_{j}-h_{1}\right|}$$

where $\theta_{tp}=\left\{g_{1},\ldots ,g_{J},h_{1},\ldots ,h_{J}\right\}$. $g_{1}$ and $g_{j}$ are the cluster centers of the forward-view gaze zone and the mirror gaze zones, respectively. $h_{1}$ and $h_{j}$ are the head movement variable of the forward-view gaze zone and the mirror gaze zones, respectively. The loss function in the gaze points space can be minimized by keeping the similar topological structure in the physical space. The function formulation is optimized in a particular order as follows. The points of the left-side mirror and right-side mirror have higher priority. Because these points have large distances in the opposite directions, they are convenient to decide. When the cluster centers of the left-side mirror and the right-side mirror are determined, the cluster center of the rear-view mirror can be naturally assigned. At this end, the initial gaze points (the gaze points of mirror gaze zones and forward-view gaze zone) are comprised of assigned labels and can be used to refine the gaze points.

#### 3.3.5. Non-Linear Global Refinement

This paper uses the non-linear global refinement to fine-tune the output of the pre-trained model. The corrected positions of the initial gaze points have undergone an unknown transformation relative to the initial values. Therefore, the refinement method should take care of the accuracy of all gaze zones and achieve the balance of gaze points’ adjustment. The refinement method is computed as

(18) $$\begin{array}{cc} \; & g_{x}^{refine}=f_{x}^{refine}\left(g_{x}\right)\\ \; & g_{y}^{refine}=f_{y}^{refine}\left(g_{y}\right) \end{array}$$

where $g_{x},g_{y}$ are the initial gaze points in the horizontal and vertical directions, respectively. $g_{x}^{refine},g_{y}^{refine}$ are the corresponding labeled gaze points. $f_{x}^{refine}\left(\cdot \right)$ and $f_{y}^{refine}\left(\cdot \right)$ are the non-linear functions of the gaze points. In its simplest form, the non-linear function model can be $f^{refine}\left(\cdot \right)=\omega f\left(\cdot \right)+\nu$, where ω and ν are function parameters.

## 4. Experiments and Discussions

In this section, we experiment with several pre-trained models to validate the generalization ability of the proposed system. Firstly, the driving data collection under naturalistic conditions is introduced. Then, the pre-trained model and baseline method for comparisons is given. Subsequently, evaluations of the proposed system are performed. Finally, an ablation study is given on within-subject evaluation and cross-subject evaluation, and error analysis is carried out on gaze estimation accuracy.

### 4.1. Naturalistic Data Collection

To evaluate the proposed system, a dataset for the field study is collected in the naturalistic driving environment. The face camera was fixed to the bottom of the windshield and the scene camera was mounted behind the driver. The images captured from the face camera are 720P high-quality images. The frame rate of the camera video is 30FPS.

**Figure 3:**
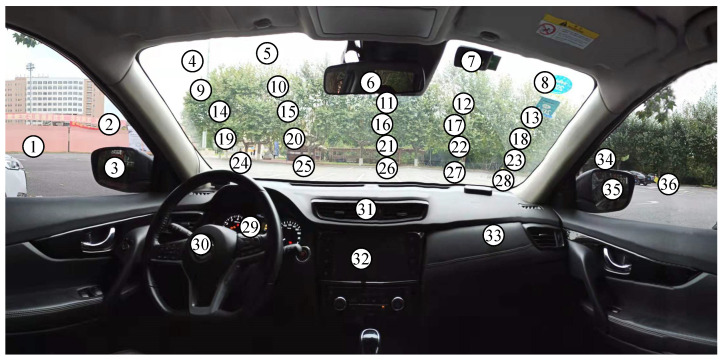
Target gaze zone partition of the on-the-road area. Gaze zone 1–36 are the target regions in the front of the driver.

The gaze region in front of the driver is partitioned into 36 gaze zones, as shown in Figure 3. All gaze zones are utilized as an indicator of the coarse gaze directions. The partitioned gaze zone covers all possible gaze regions in front of the driver to increase the variance of the head and eye status. The standard data of the driver’s gaze in different gaze zone were collected in advance to verify the manual calibration gaze error.

To facilitate the data collection, yellow markers were pasted into the center of every gaze zone, which can easily be noticed in the vehicle cabin. The drivers who participated were asked to look at the marker one after another during the calibration procedure. In the training and testing data for calibration, the dataset retains five driver subjects’ data. For each subject, the dataset has 720 annotated gaze zone data to train the model, and another 720 data to test the model. This means 20 annotated data remain for each gaze zone.

**Figure 4:**
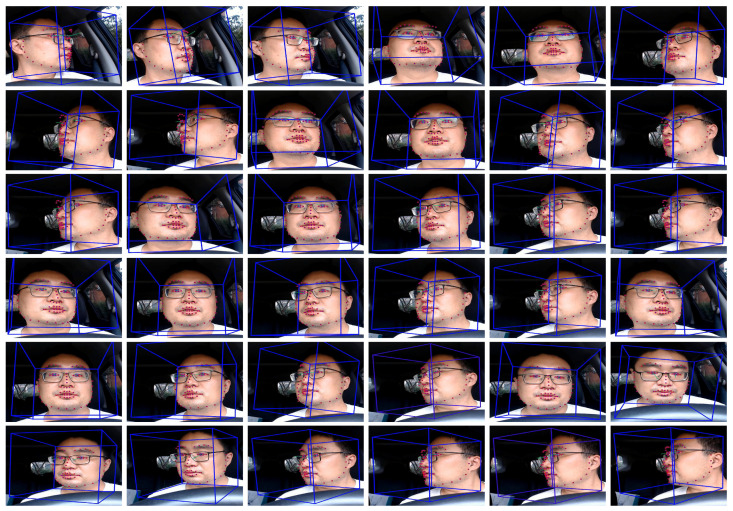
The driver status extraction results of all the partitioned gaze zone. The first row shows the results of GZ1∼GZ6. The second row shows the results of GZ7∼GZ12. The third row shows the results of GZ13∼GZ18. The fourth row shows the results of GZ19∼GZ24. The fifth row shows the results of GZ25∼GZ30. The last row shows the results of GZ31∼GZ36. GZ: Gaze Zone.

In this dataset, we adopted OpenFace [32] for convenient driver head and eye status extraction. OpenFace tools achieved stable and reliable performance on the head and gaze estimation in the collocated video compared with other existing tools. Advances in this area can benefit the driver gaze analysis and develop more robust solutions [33,34]. Figure 4 shows the face and eye detection results of different gaze zones.

### 4.2. Pre-Trained Models and Baseline Methods

In addition to the dataset, we evaluated the proposed method with the common pre-trained model, Gaussian process for regression (GPR) [23,35], partial linear square regression (PLSR) [14], non-linear square regression (NLSR). All these models were individually trained on the training dataset for different drivers. The GPR method builds the probabilistic model by taking the prior of the function space as the Gaussian process and carries out the solution according to the Bayesian inference. Here, the parameters of GPR are similar to Ref. [9]. The PLSR method trains the regression model on the principle component by projecting the variables into new space. Here, the components’ value of the PLSR model is 4, which is the same as that of the calibration gaze zones. Inspired by Ref. [5], a single NLSR model is utilized for non-linear estimation to link the inputs and output. For each driver, we trained the GPR model, PLSR model, and NLSR model for pre-trained model evaluation. Here, several calibration methods are used as the baseline method for gaze prediction comparisons, including Gaussian process mapping (GPM), homography transform projection (HTP). The GPM method builds the mapping relationship as performed in Ref. [9], and the HTP method treats the gaze point refinement as a homography transform from the primary image plane to the final image plane.

Here, the experiments are conducted on within-subject evaluation, cross-subject evaluation, and normal evaluation. Within-subject evaluation means that testing and training are performed on the same driver subject’s data and the total number of experiments is 5(subject)×1(experiment/subject)=5(experiments). While the cross-subject evaluation means that training on one driver subject’s data and testing on another driver subject’s data, the total number of experiments is 5(subject)×4(experiment/subject)=20(experiments). For the normal evaluation without specific instructions, the testing is on all driver subject’s data and the total number of experiments is 5(subject)×5(experiment/subject)=25(experiments).

### 4.3. Gaze Point Prediction Results

This section compares the proposed method with the baseline methods on the pre-trained models. The dataset consists of the driver status for each gaze zone, with labels of pixel-level gaze points and annotations of the gaze zone index. The locations of the gaze zones are labeled on the image coordinate axis. For the particular driver, the proposed method will gradually revise the gaze points and build the refinement of the gaze model.

The gaze estimation error is calculated in the perspective of the direction angle view and image pixel view. The ground-truth values are provided by the labeled anchors of gaze zone centers. The size of the scene image is 2200×1080, and the relevant angle of the full field-of-view is $137.5^{\circ }\times 67.5^{\circ }$. All the gaze estimation methods are measured by the absolute mean error of both the horizontal and vertical directions of eye gaze.

Table 1 presents the absolute mean error on a different calibration method. The proposed method achieves better results with a more than 1.4 degree error drop on all pre-trained models. Among them, the HTP method has a relatively large estimation error which may establish incorrect mapping in all possible solutions of the image plane. The HTP method can especially not obtain an output on several GPR model evaluations. At this point, the mean error of the HTP method is computed on the positive solution. The proposed method with the PLSR model has the best gaze estimation accuracy and largest accuracy improvement.

**Figure 5:**
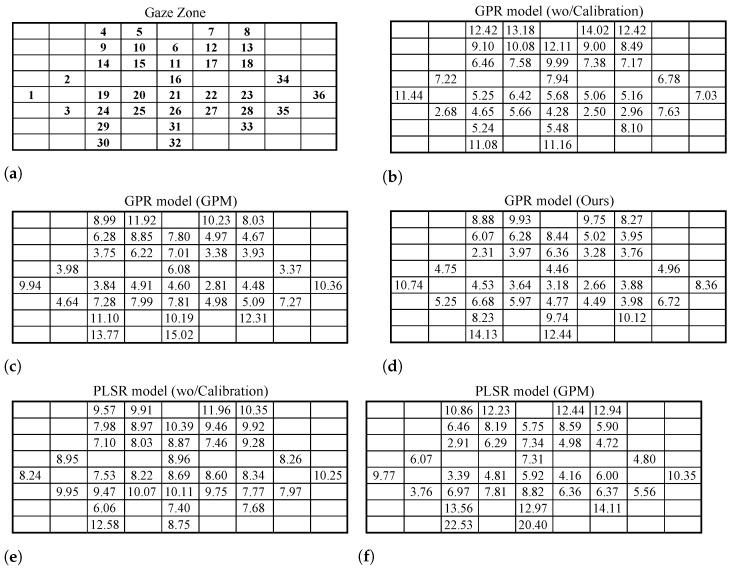

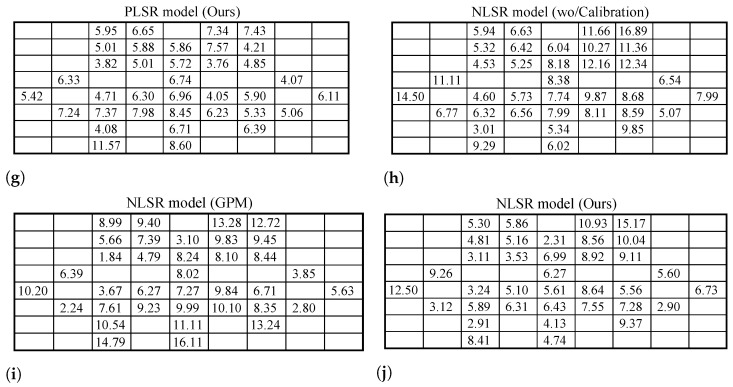
Absolute mean error (degree) on different gaze zones: (**a**) gaze zone partition; (**b**) GPR model without calibration; (**c**) GPR model with GPM method; (**d**) GPR model with the proposed method; (**e**) PLSR model without calibration; (**f**) PLSR model with GPM method; (**g**) PLSR model with the proposed method; (**h**) NLSR model without calibration; (**i**) NLSR model with GPM method; and (**j**) NLSR model with the proposed method. Compared with the related pre-trained models without calibration, the pre-trained models with GPM method or the proposed method have reduced the estimation error on gaze zones.

Figure 5 gives the absolute mean error for each gaze zone. The proposed method decreases the estimation error on more than two-thirds of the gaze zone. Among them, the error distribution of each gaze zone is relatively flat on the results of the PLSR model. In this table, the HTP method is ignored due to the unstable results on each gaze zone. Although the error decrease level varies among gaze zones, all gaze zones adjacent to the calibration gaze zones achieve a lower gaze prediction error.

### 4.4. Ablation Study and Error Analysis

This section studies the effect of various gaze zone calibration data. Table 2 gives the ablation study results calibrated with different gaze zone data. It shows that the calibration method without the right-mirror gaze zone data has the largest estimation error. This suggests that the right-mirror gaze zone is important in the calibration data since it is the only calibrated gaze point at the right side. The proposed method with scattered calibration points outperforms the method with concentrated calibration points and significantly reduces the prediction error.

Table 3 and Table 4 show the within-subject performance and cross-subject performance of the proposed method. As can be observed, all methods have poorer performance on the cross-subject evaluation than on within-subject evaluation. The proposed method can reduce the gaze estimation error in both cross-subject and within subject evaluations. There is no significant difference on the results of the proposed method with the GPR model. The mean error on cross-subject evaluation is less than 7 degrees. The gaze error of the proposed method on within-subject evaluation using the PLSR model is approximately five degrees. In the driving scenarios, the gaze tracking system has a somewhat large error tolerance, which is sufficient.

**Figure 6:**
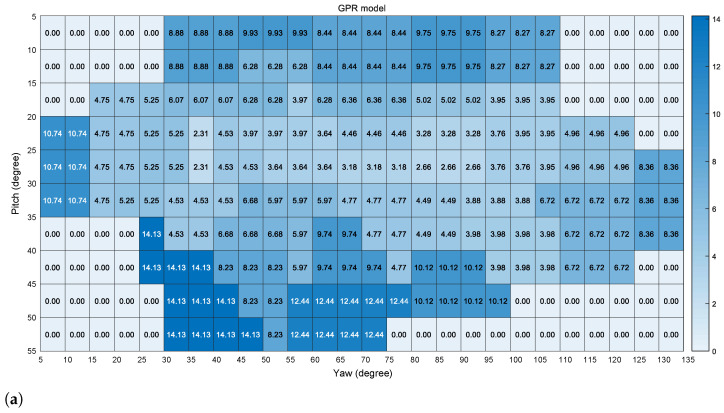

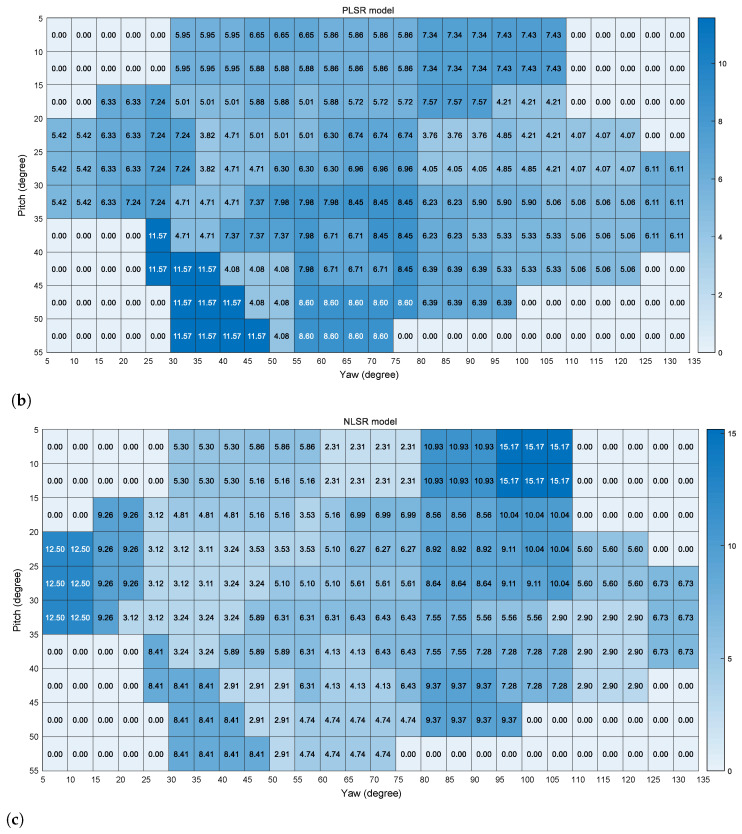
Error reduction of the pre-trained model in the horizontal and vertical directions of the image plane, the proposed method with (**a**) GPR model; (**b**) PLSR model; and (**c**) NLSR model. Each block is a square with angles of 5×5. The color of the block indicates the increase level of the gaze estimation accuracy.

In addition, the error analysis on the horizontal and vertical directions of the image plane is given for further validation. As shown in Figure 6, all pre-trained models benefit from the proposed method, and have a large error reduction after gaze point refinement. In Figure 6, each block represents a five degree angle in the horizontal and vertical directions of the image plane. The difference values with the positive effect of the pre-trained model with the proposed method are denoted by colors. Based on this statistic analysis, the gaze estimation error of the three mirror gaze zone is significantly decreased among consecutive gaze zones, whose maximum accuracy improvement can reach 15 degrees. This is effective for driver gaze refinement.

## 5. Conclusions

This paper implements a gaze tracking and mapping system for monitoring driver eye gaze using a face camera and a scene camera. The proposed system applies a non-linear gaze point refinement mechanism to automatically facilitate the gaze prediction of the pre-trained model. Extensive experiments show the potential usage of several gaze models, and give the effectiveness of the proposed method. Future works should further investigate the impact of gaze estimation variance in long-term experiments, and apply it in the deep learning-based gaze estimation method.

## Figures and Tables

**Table 1:** Absolute mean error (degree) on different calibration methods and pre-trained models. GPM: Gaussian process mapping, HTP: homography transform mapping.

Calibration Method	GPR Model	PLSR Model	NLSR Model	Average
wo/Calibration	7.74	8.97	8.09	8.27
GPM	7.16	8.37	8.20	7.91
HTP	17.98	17.63	16.99	14.45
Ours	6.39	6.13	6.59	6.37

**Table 2:** Absolute mean error (degree) of gaze estimation performance on different calibrated gaze zones. wo/means without the calibration data from the given gaze zone. Front: GZ14; Left: GZ3; Middle: GZ6; Right: GZ35; wo/Calibration: without any calibration.

Calibration Method	GPR Model	PLSR Model	NLSR Model	Average
wo/Front	8.71	6.26	6.78	7.25
wo/Left	6.70	5.96	6.62	6.42
wo/Middle	7.00	6.50	6.80	6.77
wo/Right	11.09	9.22	7.21	9.17
Ours	6.39	6.13	6.59	6.37

**Table 3:** Absolute mean error (degree) of within-subject performance on different calibration methods. GPM: Gaussian process mapping; HTP: homography transform mapping.

Calibration Method	GPR Model	PLSR Model	NLSR Model	Average
wo/Calibration	6.99	5.26	5.67	5.97
GPM	6.77	8.13	7.30	7.40
HTP	7.49	10.79	5.67	7.98
Ours	6.19	5.04	5.67	5.63

**Table 4:** Absolute mean error (degree) of cross-subject performance on different calibration methods. GPM: Gaussian process mapping, HTP: homography transform mapping.

Calibration Method	GPR Model	PLSR Model	NLSR Model	Average
wo/Calibration	7.93	9.90	8.69	8.84
GPM	7.26	8.43	8.45	8.05
HTP	20.78	19.34	19.81	19.98
Ours	6.44	6.40	6.82	6.55

## Data Availability

Not applicable.
